# 

**Published:** 1893-02

**Authors:** 


### Southern Medical Record

A Monthly Journal of Medicine and Surgery.

VOL. XXIII. ATLANTA, GA., FEBRUARY, 1893. NO 2.

EDITORS:

A. W. GRIGGS, M. D. WILLIS F. WESTMORELAND, M. D.

J. McFADDEN GASTON, M. D. DAN. H. HOWELL, M. D., BUS. MGR.

Entered at the Postoffice at Atlanta, Ga., as Second-Class Matter.

Mutual Printing Company, Publishers, 27 East Hunter St., Atlanta, Ga.

